# Using Mahalanobis Distances to Investigate Second Dialect Acquisition: A Study on Quebec French

**DOI:** 10.1177/00238309221097978

**Published:** 2022-06-20

**Authors:** Josiane Riverin-Coutlée, Johanna-Pascale Roy, Michele Gubian

**Affiliations:** Département de langues, linguistique et traduction, Université Laval, Canada; Institute of Phonetics and Speech Processing, Ludwig-Maximilians-Universität München, Germany; Département de langues, linguistique et traduction, Université Laval, Canada; Institute of Phonetics and Speech Processing, Ludwig-Maximilians-Universität München, Germany

**Keywords:** Second dialect acquisition, Quebec French, acoustics of vowels, longitudinal data, Mahalanobis distances

## Abstract

Second dialect acquisition (SDA) can be defined as the process through which geographically mobile individuals adapt to new dialect features of their first language. Two common methodological approaches in SDA studies could lead to underestimating the phonetic changes that mobile speakers may experience: only large phonetic differences between dialects are considered, and external sources are used to infer what should have been the speakers’ original dialect. By contrast, in this study, we carry out a longitudinal analysis to empirically assess the speakers’ baseline and shift away from it with no priors as to which features should change or not. Furthermore, we focus on Quebec French, a variety with a relatively crowded vowel space. Using Mahalanobis distances, we measure how acoustic characteristics of vowels produced by 15 mobile speakers change relative to those of a control group of 8 sedentary speakers, with the mobile participants recorded right after they moved to Quebec City, then a year later. Overall, the results show a reduction of Mahalanobis distances over time, indicating convergence toward the control system. Convergence also tends to be greater in denser areas of the vowel space. These results suggest that phonetic changes during SDA could be finer than previously thought. This study calls for the use of methodological approaches that can reveal such trends, and contributes to uncovering the extent of phonetic flexibility during adulthood.

## 1 Introduction

Individuals are increasingly mobile. While larger cities, where economic opportunities abound, have always attracted a fair number of newcomers, the motivations, profiles, and routes of migration have never been so diverse (see Vertovec, 2007). This, of course, has a potentially significant impact on the linguistic landscape of a place that attracts migration, but also on the linguistic practices of the mobile individuals themselves. Leaving aside those situations where a new language needs to be learned (second language acquisition [SLA]), mobile speakers are often faced with new features of their first language. Usually called *second dialect acquisition* (SDA), the adaptation to various degrees to these new features has recently arisen as a full and legitimate topic (see Nycz, 2015; Siegel, 2010 for reviews).

SDA can take place at all linguistic levels (phonetic, phonological, morpho-syntactic, etc.), but the most studied aspect is undoubtedly pronunciation (Auer, 2007), which is also the focus of the current article. As the number of studies investigating phonetic and phonological aspects of SDA increased, it soon became clear that heterogeneity was the rule, with some speakers barely adapting while others do so more extensively, some features being quickly adopted and others lagging behind (e.g., Munro et al., 1999). Several factors have been put forward to account for this, some of them social in nature, as well as linguistic and cognitive: the speakers’ integration and involvement in the new community (De Decker, 2006; Pesqueira, 2008), age (Hazen & Hamilton, 2008), regional identity (Campbell-Kibler et al., 2014; Evans & Iverson, 2007), attitudes toward the second dialect (Bigham, 2010; Sprowls, 2014) and interlocutors (Giles, 1973); the linguistic level and complexity of a feature (Chambers, 1992; Kerswill, 1996; Rys & Bonte, 2006; Vousten & Bongaerts, 1995); the speakers’ linguistic sensitivity (Nycz, 2013), degree of attention to the communication situation (Sharma, 2018), and robustness of their representations (Reubold & Harrington, 2018); and so on.

However, in the long term, understanding how SDA happens and why it is so heterogeneous is hampered by the focus of most studies on the outcome of SDA as either a complete success or failure, that is, “a matter of all or nothing” (Siegel, 2010, p. 138). Most studies also infer from external sources what should have been the speakers’ original dialect (Auer et al., 1998; Bigham, 2010; Campbell-Kibler et al., 2014; Conn & Horesh, 2002; Evans, 2004; Foreman, 2003; Hazen & Hamilton, 2008; Hernández & Maldonado, 2012; Ivars, 1994; Miller, 2005; Munro et al., 1999; Nuolijärvi, 1994; Omdal, 1994; Sprowls, 2014; Ziliak, 2012; etc.); therefore, *changes* (or absence thereof) can only be presumed. Another issue is that most studies of SDA focus exclusively on major, perceptible, and discrete regional differences. Picking up on authors like Trudgill (1986), Siegel (2010, p. 133) even argues that “in order to be acquired, a variant must be salient enough to be noticed.”^1^ Methodologically speaking, these are no-nonsense approaches and they are not unrelated. Following individuals longitudinally is a notoriously difficult and costly task (Gerstenberg & Voeste, 2015; Sankoff, 2018a), not to mention the logistic challenge of recruiting speakers who are about to move or have just done so (Nycz, 2015). Concentrating on large differences allows researchers to quickly tap into phonetic characteristics that mobile speakers are likely to have changed, and in the absence of longitudinal data, might be the only way of investigating SDA. However, the full extent and nature of changes, especially those happening in early stages of SDA, thereby remain a blind spot.

This strikes as even more problematic when moving onto studies addressing phonetic convergence (or imitation or accommodation), a field closely related to SDA. Convergence can be defined as “an increase in similarity among linguistic components” of interacting individuals’ productions (Pardo, 2006, p. 2383). Typically, in studies on phonetic convergence, speakers interact in a lab setting and the degree to which the phonetic properties of their speech change as a result of this short-term interaction is investigated (e.g., Pardo, 2006). Since these studies focus on speech recorded *throughout* the experiment, any shift from a speaker’s baseline can be tracked, and (un)expected shifts have indeed been tracked (see Coles-Harris, 2017 for a review). They also suggest that the necessity for a feature to be salient to trigger convergence is not as absolute as previously thought: convergence toward non- or less salient features (Babel, 2010; Delvaux & Soquet, 2007; MacLeod, 2014; Pardo et al., 2012) and even divergence from salient features (MacLeod, 2014) have been documented. Based on these insights from work on convergence, the question is: Is SDA assumed to occur only on large phonetic differences because only large phonetic differences have been investigated due to methodological constraints?

In this paper, we intend to fill a part of this research gap, that is, to explore initial stages of the process of SDA through an empirical assessment of the speakers’ baseline and potential shift away from it, regardless of the size of phonetic differences that there might be with the target variety. Specifically, we investigate (acoustic) phonetic changes taking place among mobile speakers of Quebec French;^2^ drawing from the convergence framework, the data is longitudinal, with speakers recorded twice over a year and compared to a control group. We do not focus on specific features known to be regional differences, but examine whether and how acoustic properties of 14 vowels change over time. This latter approach is also motivated by practical reasons, as modern regional differences in Quebec French are not well documented (see Section 2).

This very first attempt, at least to our knowledge, at studying SDA in Quebec French also provides an opportunity to diversify the linguistic contexts in which this process is investigated and reflected upon. Beyond the descriptive interest of such a diversification, it is crucial to improve our methodological approaches, theoretical knowledge, and models of SDA. First, as will be explained in Section 2, Quebec French has a relatively crowded vowel space (Maddieson, 1984; Schwartz et al., 1997), which is thought to constrain variation in the articulation and acoustics of vowels compared to less crowded systems, partly because perceptual differences have to be preserved (e.g., Lindblom, 1986; Manuel, 1990). In parallel, some researchers have argued that speakers may use a combination of convergence and non-convergence in order not to impede phonological contrasts (e.g., Nielsen, 2011; Wagner et al., 2021). Although it is not our purpose to test specific predictions related to this, our study may nonetheless offer some insights on the process of SDA in a crowded vowel space, and also put forward the possible use of Mahalanobis distances (MDs; Mahalanobis, 1936) in such a context, a metric that is sensitive to properties of multidimensional data distributions like those of vowels in the formant (F1 × F2) space (see Section 3.3).

Second, early work mainly framed SDA within the speech or communication accommodation theories (Giles, 1973; Giles et al., 1991). Trudgill (1986) proposed that an accumulation of short-term accommodation led to long-term accommodation, or SDA. However, such models which attribute convergence and change to social, psychological, and attitudinal macro-factors are not known for their focus on linguistic details. More recently, exemplar-based models (e.g., Foulkes & Docherty, 2006; Johnson, 1997; Pierrehumbert, 2006) have been brought about by several authors as an explanation to the process of SDA (e.g., Love & Walker, 2013; Nycz, 2013; Reubold & Harrington, 2018; Rys et al., 2017; Walker, 2018). Models of this kind suggest that listeners memorize in great detail incoming linguistic events. These are eventually grouped into categories based on details that are relevant, frequent, and recent, and speakers-listeners can use these details and categories to either classify new incoming linguistic events or to produce events themselves. Features of a second dialect are acquired because they have been frequently and recently heard, and because they are relevant to the situation. Accommodation and exemplar theories are compatible to a certain extent (Coles-Harris, 2017), that is, they are both adequate to explain large phonetic shifts motivated by indexical factors, but exemplar-based models predict and account for much finer shifts than accommodation theories. If our results reveal small phonetic shifts, this would not necessarily provide evidence against accommodation theories, but would support exemplar-based models.

In sum, our aim is to import methods and principles from the convergence paradigm into a study of SDA where the variety under investigation has a crowded vowel space, to offer a new, more nuanced perspective on the size of the phonetic shifts that may occur in early stages of SDA, which will contribute to our understanding and modeling of the phenomenon. The paper is organized as follows: in Section 2, we will first provide readers with some essential indications regarding Quebec French phonetics; the methods used in this study will then be detailed in Section 3 and the results obtained will be presented in Section 4; we will use Sections 5 and 6 to discuss and put our results into perspective.

## 2 Quebec French phonetics

Quebec French is described as comprising the 38 phonemes listed below (inventory based on Martin, 1996; Martin et al., 2001; Riverin-Coutlée & Roy, 2020). This makes it a system with a large number of vowels, as well as a large vowel-to-consonant ratio, compared to other languages of the world (Maddieson, 1984).

- 18 consonants: /p t k b d ɡ f s ʃ v z ʒ l ʁ m n ɲ ŋ/
- 3 glides: /j ɥ w/
- 17 vowels: /i y u e ø o ə ɛ œ ɔ a ɑ a͜ɛ ã ɔ̃ ẽ œ̃/

In this paper, we exclusively focus on phonemic monophthongal oral vowels that can be stressed, for which the following indications appear necessary. First, schwa is never stressed, and there is only little evidence that it is acoustically distinct from /œ/ (Martin, 1998), so we have merged it with /œ/ here. Second, in word-final syllable, the high vowels /i y u/ split into mutually exclusive tense and lax variants, [i y u] versus [ɪ ʏ ʊ], depending on the consonantal context. The former, [i y u], appear in open syllables (V#) and when followed by one of the lengthening consonants /v z ʒ/ or /vʁ/ (e.g., *vie* [vi] “life,” *luge* [lyʒ] “sleigh”). The lax variants [ɪ ʏ ʊ] are produced when followed by the remaining consonants or glide /j/ (e.g., *fille* [fɪj] “girl,” *poule* [pʊl] “hen”).^3^ Thereby, our study is based on the following 14 vocalic categories: /i ɪ y ʏ u ʊ e ø o ɛ œ ɔ a ɑ/. Note that all vowels, including the tense and lax variants of the high vowels, will be enclosed within slashes throughout the paper for consistency.

The issue of geographical phonetic variation in Quebec French has predominantly been addressed in a comparative perspective with European varieties of French (e.g., Côté, 2012; Dumas, 1987; Gendron, 1966). Very little is currently known about regional variation *within* Quebec (Dolbec & Ouellon, 1999), and despite some progress in recent years, the phonetic landscape of the province is far from being defined, especially the geographical distribution of the phonetic features that have been identified as modern-day regional specificities. For example, Remysen (2016) noticed a very backed /ɑ̃/ in Montreal as opposed to Sherbrooke, but his data are limited to speakers from Montreal and Sherbrooke. Similarly, Riverin-Coutlée & Arnaud (2014) found that word-final /ɛ/ was lowered in Saguenay as compared to Quebec City, but did not consider neighboring regions such as Charlevoix, which holds strong historical and linguistic ties with Saguenay (Dolbec & Ouellon, 1999). In other words, Quebec French does vary regionally, so there might be larger phonetic differences some mobile speakers may be prone to modify, but since we do not exactly know which ones and by whom, a less targeted approach to studying SDA was needed.

## 3 Methods

### 3.1 Speakers

Fifteen native speakers of Quebec French, all females aged 18–21 years, were recruited upon enrollment in an undergraduate program at Université Laval, in Quebec City (Quebec, Canada). The first experiment (T1) took place in September 2016, in the initial weeks after they started university. They were from 13 different towns and cities around the province^4^ and will henceforth be referred to as the mobile speakers. When first tested, 13 of them had been living in Quebec City for less than a month, 1 for 3 months (Mobile11) and 1 for a year (Mobile03). Otherwise, the mobile speakers had always lived where they were born, except for short intervals (e.g., a 6-month student exchange in South America to learn Spanish). A replication of the experiment was set up in September 2017 (T2), as the participants were starting their second year at Université Laval and had been living in Quebec City for a year (or two, for Mobile03).

At T2, an additional eight female speakers were recruited for comparison. They were also native speakers of Quebec French aged 19–22 and were starting their second year at Université Laval, but they were sedentary. They had always lived in Quebec City or Levis (the south shore), except for one speaker who was born in the United States to French-speaking parents and had arrived in Quebec City at age 1. They were recruited through an email sent to all undergraduate students at Université Laval, and may or may not have known some of the other 15 participants. They performed the same tasks as the mobile speakers.

The participants were blind to the subject of the experiment, that is, they were only informed at the end of T2 that SDA was the topic of investigation. They received a $10 compensation after each participation.

### 3.2 Speech material

The participants were recorded individually in a sound-attenuated booth at Université Laval, using a Tascam DR-100 MKIII (digital format, 44 100 Hz, 16 bits). They took part in three reading tasks that were identical for both experiments and both groups of speakers. In Task 1, they were presented with meaningful though out-of-context carrier sentences that ended with a target word (see Appendix A). For example, the following sentences featured the target words *drôle* /dʁol/ “funny,” *jeune* /ʒœn/ “young” and *soupe* /sʊp/ “soup,” respectively:

- Cette fille est drôle. “This girl is funny.”
- La soirée est encore jeune. “The night is still young.”
- Boucles d’or goûte à la soupe. “Goldilocks tastes the soup.”

The lexical items used for both the carrier sentences and target words correspond to a standard but common register in Quebec French, although we did not explicitly control for lexical frequency. The sentences were presented randomly, but the order was identical for all participants.

In Task 2, the same target words as in Task 1 were inserted in a fixed carrier sentence:

- Je pense au mot *drôle* très fort. “I am thinking very hard about the word *funny*.”
- Je pense au mot *jeune* très fort. “I am thinking very hard about the word *young*.”
- Je pense au mot *soupe* très fort. “I am thinking very hard about the word *soup*.”

Task 3 featured the same target words in isolation:

- Drôle “Funny”
- Jeune “Young”
- Soupe  “Soup”

The motivations for designing three tasks were to avoid a task effect and to ensure that excessive repetitiveness would not cause the mobile speakers to drop off from the experiment after T1. The participants were shown one sentence or word at the time on the screen of an electronic tablet at a pace controlled by the experimenter. They were told that they were not judged on performance, so that they should read naturally and feel free to start over any sentence they felt had been misread, for example, when skipping a word. Unless an external noise impeded recording quality, the experimenter avoided asking the participants to read over any sentence, even if the target word was not produced as expected (in particular, orthographically similar *jeune* /ʒœn/ and *jeûne* /ʒøn/ were frequently confused in the absence of a meaningful sentence, i.e., in Tasks 2 and 3), to maintain a relaxed atmosphere. A short training phase helped the participants familiarize with the three tasks.

Seventy words uttered during each task are analyzed here, as they comprise one of the 14 vowels considered in one of eight possible phonetic contexts: followed by a voiced or voiceless stop (/b d ɡ/ or /p t k/), by a nasal stop (/m n/), by a voiced or voiceless fricative (/v z ʒ/ or /f s ʃ/), by the lateral /l/, by the rhotic /ʁ/, and in open syllables. Not all combinations of vowels and contexts yield French words, for example, /o/ does not appear in a syllable closed by /ʁ/ while /e/ only appears in open syllables. In total, 7,980 vowels were available for the analyses described in the next section (i.e., 70 vowels × 3 tasks × 2 experiments × 15 mobile speakers, plus 70 vowels × 3 tasks × 1 experiment × 8 sedentary speakers).

### 3.3 Analysis

The recorded speech was analyzed acoustically. Using *Praat* (Boersma & Weenink, 2020), the vowels were first segmented manually by a trained acoustician. The following cues were used to establish vocalic boundaries: from periodicity onset to offset, from a rise to a drop in intensity and from lower formants’ appearance to disappearance. Acoustic cues from adjacent consonants were also exploited, for example, bursts, high-frequency noises, voice bars, as well as auditory cues. Nineteen tokens were discarded at this stage because they were impossible either to segment or to analyze afterward, most of them high vowels that underwent devoicing in voiceless consonantal environments, a common phenomenon in Quebec French (Dumas, 1987; Paradis & Dolbec, 1998). Frequency of the first two formants (F1 and F2) was then measured at vowel midpoint. Formant settings were manually set for each speaker and each vocalic category to optimize detection. When needed, the formant detection ceiling and number of formants to be detected in the frequency interval were adjusted per token. In the end, 15,922 formant measurements were gathered.

We based our evaluation of whether mobile speakers have converged toward Quebec City speech over time on MDs (Mahalanobis distances; Mahalanobis, 1936), in the acoustic space, between the tokens they produced at T1 and T2 and those from the sedentary speakers. Specifically, the acoustic space is the F1 × F2 plane, where each token corresponds to a single point. The tokens from the sedentary speakers were used to estimate reference distributions for each of the 14 vowels. These distributions were characterized by their location (centroid) and spread parameters, the latter in the form of a 2 × 2 covariance matrix, which accounts for both spread and correlation between F1 and F2 values. This level of description amounts to assuming that formant values within each vowel category are distributed as two-dimensional Gaussians, a fairly common assumption in the literature (Whalen & Chen, 2019). We then computed the MD between each token of a given vowel category produced by the mobile speakers and the corresponding reference (sedentary) distribution using formula B1 in Appendix B. The reason for preferring Mahalanobis to the better-known Euclidean distances^5^ is that the former take into account not only the centroid location, but also the spread and orientation of the reference distribution.

**Figure 1.**
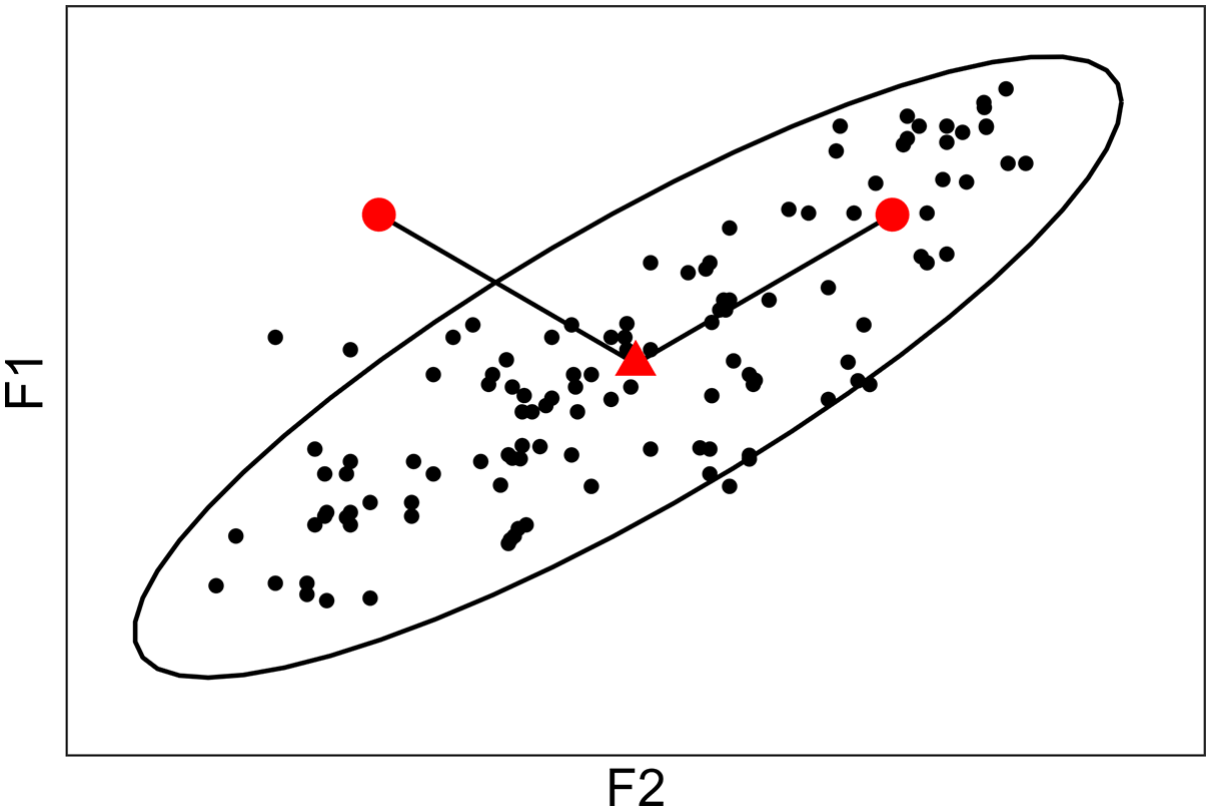
Distance between the centroid (red triangle) of a hypothetical distribution and two given datapoints (large red dots). F1 and F2 are positively correlated; F2 has large variance and F1 has small variance.

To better illustrate this point, a plausible distribution of vocalic tokens in an F1 × F2 plane is presented in Figure 1. Note that the ellipsoidal shape of the distribution reflects the hypothesis of Gaussianity mentioned above. In this plot, the two large red datapoints are equidistant from the centroid of the distribution, represented by the red triangle. If the Euclidean distance between the datapoints and centroid was calculated, the two results would be identical. However, it is obvious from Figure 1 that the position of the datapoints relative to the distribution is not the same: the right datapoint is within the range of plausible formant values for this particular vowel, while the left datapoint is an outlier. This is because for this particular hypothetical vowel: F1 and F2 are positively correlated; F2 has large variance while F1 has small variance. Contrary to Euclidean distances, MDs take into account variance and correlations in the data, in a way that can be understood as an extension of *z-*score normalization (Lobanov, 1971)—that is, dividing the distance to the mean of a unidimensional Gaussian distribution by its standard deviation—to the multidimensional case. In Figure 1, the datapoint that is part of the cluster therefore has a smaller MD to the centroid than the outlier. If we now imagine these datapoints shifting toward the centroid, the value of the MD covered would also depend on the relationship between the datapoints and the distribution (one absolute unit closer to the centroid is not one Mahalanobis unit closer).

**Figure 2.**
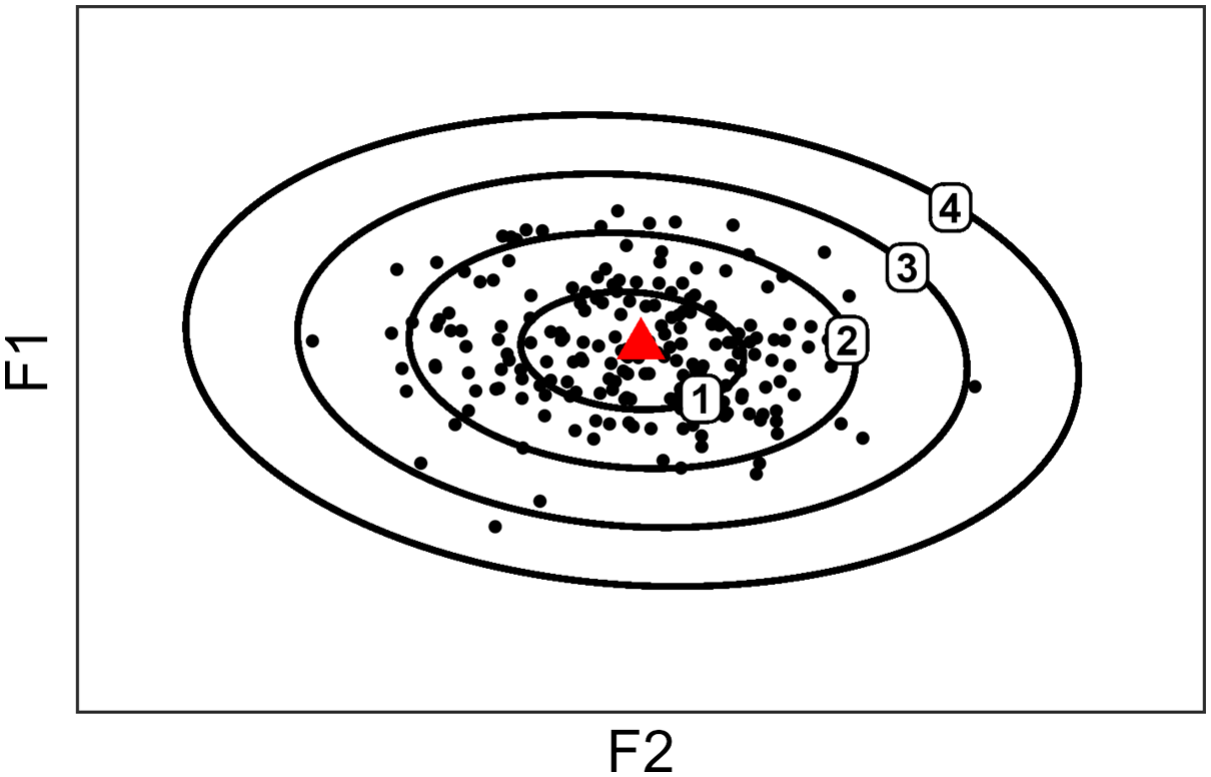
Distribution of data encompassed into four ellipses corresponding to 1, 2, 3, and 4 Mahalanobis distances away from the centroid (red triangle).

This is made explicit in Figure 2, which gives an example of possible MD values for a given distribution. Four ellipses are represented, the smallest and closest to the centroid or mean (red triangle) encloses points within a MD of 1 from the mean; the second closest encloses points within a MD of 2 from the mean; and so on. If the distribution in Figure 2 is Gaussian, then those ellipses encompass 39.35%, 86.47%, 98.89%, and 99.96% of the probability mass, respectively (see Appendix B for details).

Once the MD corresponding to each token produced by the mobile speakers was computed, a linear mixed-effects regression model was fitted to the log-transformed data (*lme4* and *lmerTest* packages in R, Bates et al., 2015; Kuznetsova et al., 2017; R Core Team, 2021). Log-transformation of non-negative continuous values like distances, durations, and so on is a common pre-processing step when such values appear as response variable in linear regression models, because it reduces the skewness of residuals, making their distribution closer to a Gaussian. In the text, though, all results will be presented in their original scale, that is, the log-distances have been converted back to distances by exponentiation. Using R formula notation, the model was as in (1)

(1) lmer(log(MD)~1+Time+(1+Time|Vowel)+(1+Time|Speaker))

where *Time* is a two-level fixed factor (T1 and T2), *Vowels* and *Speakers* are random factors with a random intercept and a random slope for *Time*. The contrast structure for the fixed effect was set to treatment coding, that is, T1 is the control level (intercept) and *Time* is the T2-T1 difference in the response variable.

Since the logic of considering speakers as a random factor is probably familiar to many readers and is well explained elsewhere (e.g., Drager & Hay, 2012), we simply mention here that it is aimed at modeling the variance of the response variable attributable to random sampling of individuals within a population. The levels of that variable (i.e., the different speakers) were not selected on purpose; there are other speakers out there, and should we recruit more, they are not expected to affect the response variable distinctly from those already considered. Setting vowels as a random effect might be less intuitive, but in this particular case, follows the same logic. We do not expect the MDs to vary in a particular manner because of the identity of the vowel, as it would be, for example, for F1 or F2. Should we add an extra vowel to the corpus (which would actually be possible, since other vowels do exist in Quebec French), we would expect its effect to fall within the range of effects already modeled, just like if an extra mobile speaker was recruited. Besides that, the regularization constraints imposed on the levels of a random factor make the estimation a great deal more efficient than the alternative of dealing with 14 independent vowel levels coded as fixed effect (see e.g., Lee et al., 2006, Sect. 5.3.1).

Estimated marginal means corrected for random effects (henceforth, estimates) were computed for each speaker and vowel across time.^6^ The intercept value was taken as the estimate at T1, while the estimate at T2 was calculated by adding the slope to the intercept. Within the current study, these estimates are used to explore how far or close certain speakers or vowels are relative to the global trends predicted by the model (see Baayen, 2008; Bates et al., 2015; Clark & Watson, 2016; Drager & Hay, 2012; Hall-Lew, 2013; etc.). As heterogeneity is a recurring observation across SDA studies, this is a way of quantifying it, at least regarding individual behaviors. It also has the potential to reveal trends for vowels (e.g., could front vowels be more prone to convergence?). A further interest of extracting estimates per vowel is that these values can be used to recreate an F1 × F2 plane where estimated MD at T1 and T2 are represented by ellipses, as in Figure 2. Readers may find out more about the implementation of the methods in R in the supplementary material.

## 4 Results

The result section is organized as follows. In Section 4.1, we briefly comment on the vowel space of the sedentary speakers, as it acts as the target of convergence. We then move on to present results from the mobile speakers in Section 4.2, first from a qualitative point of view, then quantitatively, based on the computed MDs.

### 4.1 Sedentary speakers

**Figure 3.**
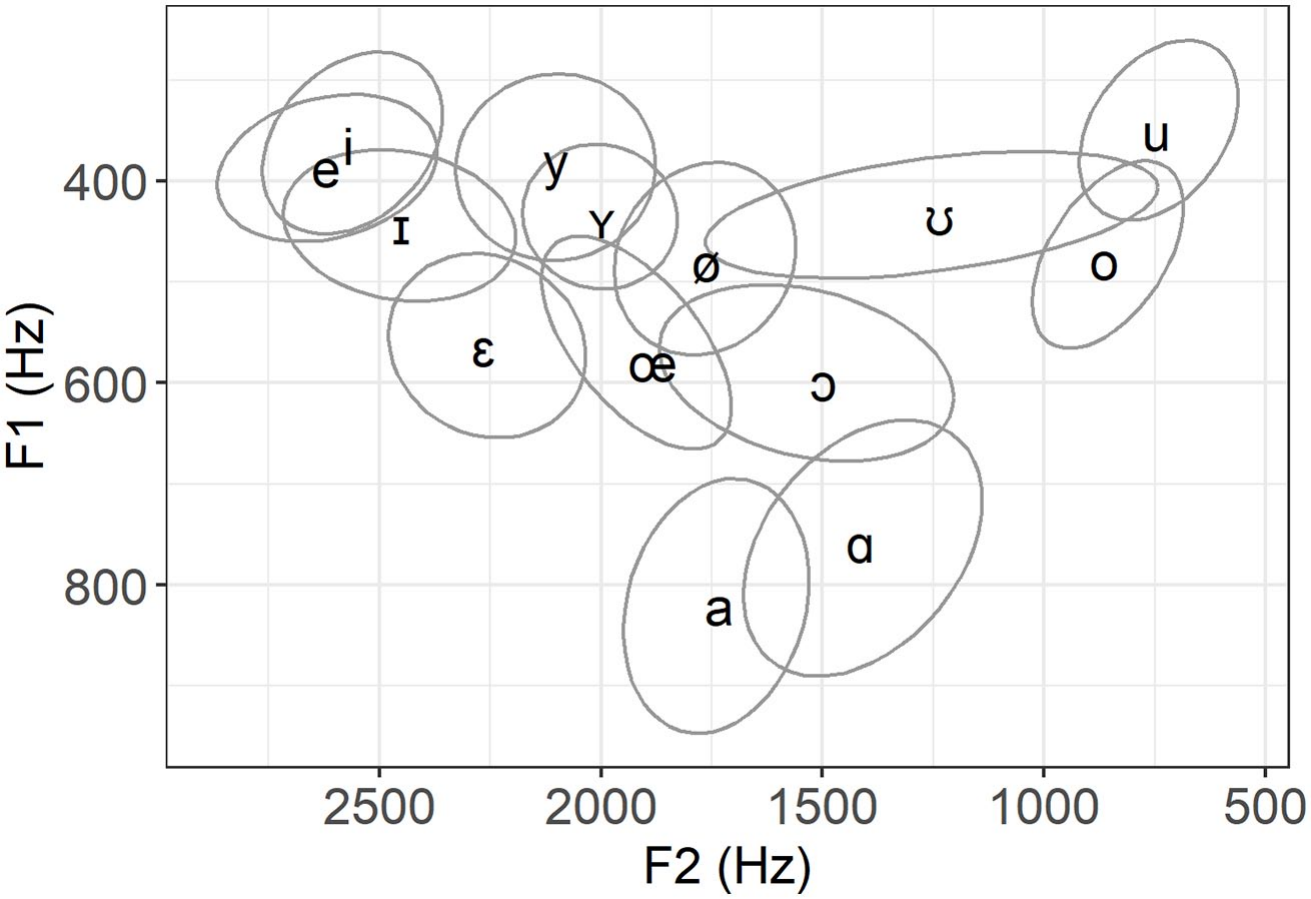
Mean vocalic system of eight sedentary female speakers at T2. The vowel labels correspond to the centroids of the confidence ellipses with alpha level of 25%.

Figure 3 and Table 1 show the target system the mobile speakers will be compared with, that is, the mean F1 and F2 values for the 14 vocalic categories produced by 8 sedentary speakers. Figure 3 only displays confidence ellipses around the mean to enhance readability, but scatter plots of all individual tokens from the sedentary speakers can be consulted in Appendix C.

**Table 1.** Mean F1 and F2 values of 14 vocalic categories produced by eight sedentary female speakers at T2.

	i	ɪ	y	ʏ	u	ʊ	e	ø	o	ɛ	œ	ɔ	a	ɑ
F1 (Hz)	364	443	380	437	351	434	387	481	478	564	579	598	821	757
F2 (Hz)	2,569	2,449	2,100	1,997	745	1,237	2,618	1,764	863	2,258	1,883	1,497	1,734	1,410

To say a few words about the F1 × F2 plane of Figure 3, we observe that the upper part of the vowel space shows more inter-category overlap than the bottom part. We have to keep in mind, however, that some vowels in the upper part might be further distinguished on other acoustic dimensions not presented here (e.g., F3, duration). The /ʊ/ variant shows evidence of fronting and is more clearly separated from its tense counterpart than the other lax variants. Similarly, /ɔ/ is centralized, a tendency previously documented in Quebec French by St-Gelais et al. (2018). The empty space between /ɛ/ and /a/ corresponds to the area where the /a͜ɛ/ phonological diphthong, not analyzed here, should be located at 50% of its rising and fronting trajectory (Leblanc, 2012; Riverin-Coutlée & Roy, 2020).

### 4.2 Mobile speakers

An overview of the longitudinal changes in the mobile speakers’ vowel system relative to the target system is presented in Appendix D, which shows F1 × F2 planes per mobile speaker. We state upfront that we observe more change than stability, and more convergence than divergence. However, before moving on to confirm these trends statistically, we use the current section to have a look at intra- and inter-speaker variability in the data. The purpose is to get away from a tendency to view SDA as a wholesale process that results in either complete success or complete failure (Siegel, 2010, p. 138). Instead, we aim at answering the following questions related to variability: are speakers categorical in whether they converge or diverge; is a given vowel always converged toward or diverged from; is the magnitude of change comparable across speakers and vowels; are there other discernable patterns of change than convergence or divergence? The figures depicted in this section are selected excerpts from Appendix D. The gray ellipses and vowel labels represent distributions and centroids of the data from the sedentary participants. The larger vowel labels correspond to the mobile speakers’ mean productions at T2 and the dots connected to the labels correspond to the mean productions at T1.

**Figure 4.**
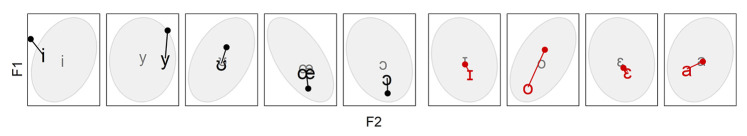
Mean value of vowels [i y ʊ oe ɔ] and [ɪ o ɛ a] produced by Mobile01 at T2 (large phonetic symbols) and T1 (dots connected to the symbols).

First, Figure 4 gives an example of intra-individual variability. It shows that a single speaker, Mobile01, has converged toward targets /i y ʊ œ ɔ/ over time, but has also diverged from /ɪ o ɛ a/.

**Figure 5.**
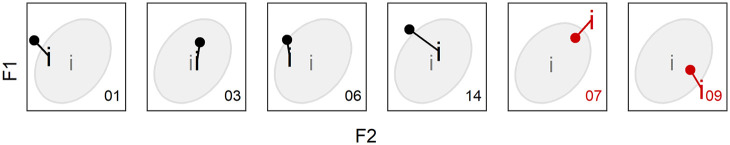
Mean value of vowel [i] produced by six different mobile speakers at T2 (large phonetic symbols) and T1 (dots connected to the symbols).

Figure 5 illustrates how a given vowel may have been both converged toward and diverged from over time. It shows that four speakers, Mobile01, Mobile03, Mobile06, and Mobile14, have converged toward target /i/, but that two speakers, Mobile07 and Mobile09, have diverged from that very same target.

**Figure 6.**
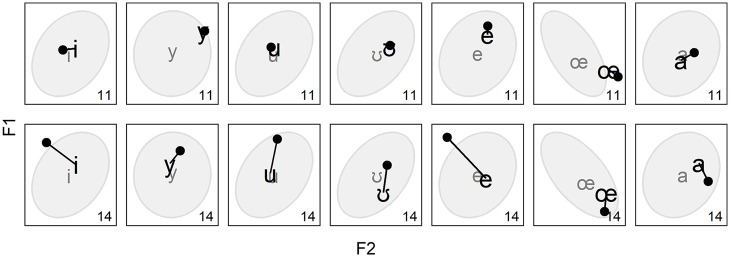
Mean value of vowels [i y u ʊ e oe a] produced by Mobile11 (top row) and Mobile14 (bottom row) at T2 (large phonetic symbols) and T1 (dots connected to the symbols).

Another qualitative observation that we make is that the magnitude of change over time varies across speakers. This is illustrated in Figure 6, which compares Mobile11, who has remained relatively stable over time, and Mobile14, who has changed more extensively, to the extent that she even ends up diverging from certain targets (e.g., /ʊ/).

**Figure 7.**
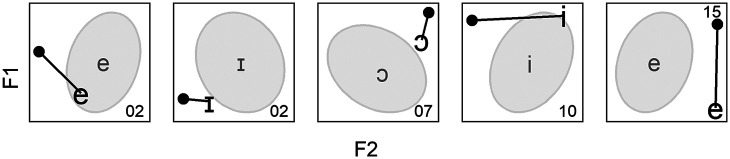
From left to right, mean value of vowels [e ɪ ɔ i e] produced, respectively, by Mobile02, Mobile02, Mobile07, Mobile10, and Mobile15 at T2 (large phonetic symbols) and T1 (dots connected to the symbols).

As can also be seen in Figures 4 to 6, as well as Appendix D, the majority of the mobile speakers’ mean productions at T1 and T2 are located within the ellipses or slightly outside. This suggests that even upon arrival in Quebec City, many of the speakers’ vowels already fell within a “locally acceptable” range. Nonetheless, Figure 7 shows examples of vowels that differ more from the local speech. Most converge toward the ellipses and centroids over time, but the rightmost panel also shows a vowel that changes a lot while staying at a great distance.

**Figure 8.**
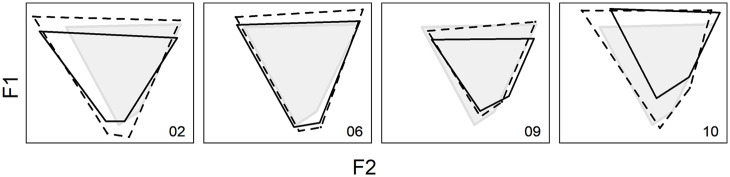
Vowel space of speakers Mobile02, Mobile06, Mobile09, and Mobile10 at T1 (dashed) and T2 (solid), and vowel space of the sedentary speakers (gray). The vowels at the four corners of the trapezoids are [i u a ɑ].

We observe for some speakers a specific pattern of change where the vowel space is reduced over time. Figure 8 presents trapezoids connecting the four cardinal vowels of Quebec French, /i u a ɑ/. The dashed trapezoids, which correspond to the mean cardinal vowels produced at T1, are larger than the solid trapezoids, connecting the mean vowels at T2. Visually, this reduction trend seems to coincide with convergence toward the target space, in gray, for Mobile02 and Mobile06, that is, these two speakers’ vowel space was slightly more expanded at T1 than the target space. For Mobile09 and Mobile10, the reduction leads to divergence from the space formed by the target cardinal vowels.

**Figure 9.**
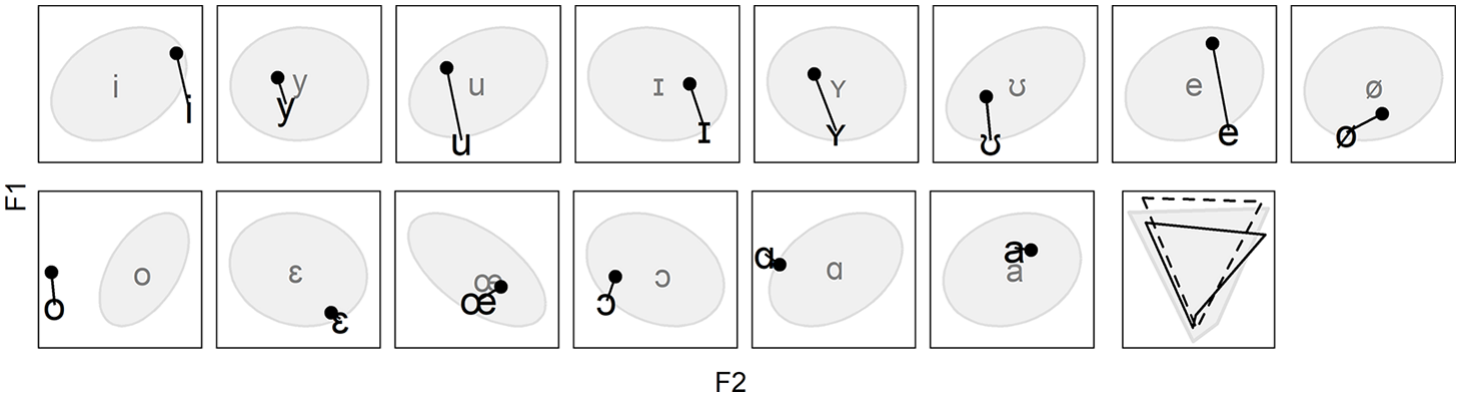
Mean value of the 14 vowels produced by Mobile05 at T2 (large phonetic symbols) and T1 (dots connected to the symbols). The bottom right panel shows the vowel space of the sedentary speakers (gray) and that of Mobile05 at T1 (black dashed) and T2 (black solid).

One last qualitative observation we make is that one speaker stands out. As depicted in Figure 9, Mobile05 has changed substantially over time, but diverged from virtually all targets, in certain cases from a close proximity at T1 (e.g., /y ɪ ʏ œ/). Yet, the changes are not random: all vowels but [a ɑ] have had their F1 raised over time, leading to an overall compression of the vowel space on the F1 axis, but not on the F2 axis (bottom right panel of Figure 9, which additionally shows the target space in gray). The vowel space of Mobile05 also has the peculiarity of being more triangular than trapezoidal, with /a ɑ/ very close to each other.

This first section on results has focused on intra- and inter-speaker variability. Through a series of selected examples, we have shown that the answer to the first three questions listed at the beginning of this section was no: speakers are not categorical in whether they converge or diverge (Figure 4), vowels can be both converged toward and diverged from (Figure 5), and the magnitude of change varies across speakers and vowels (Figures 6 and 7). The answer to the last question is yes: a reduction of the vowel space is observed for certain speakers (Figure 8) while one particular speaker seems to have changed non-randomly but independently from the target system (Figure 9). On one hand, this confirms that the heterogeneity encountered in previous work on SDA is reflected in ours. On the other hand, this suggests that there is more to SDA than complete success or failure, especially in the first stages.

**Figure 10.**
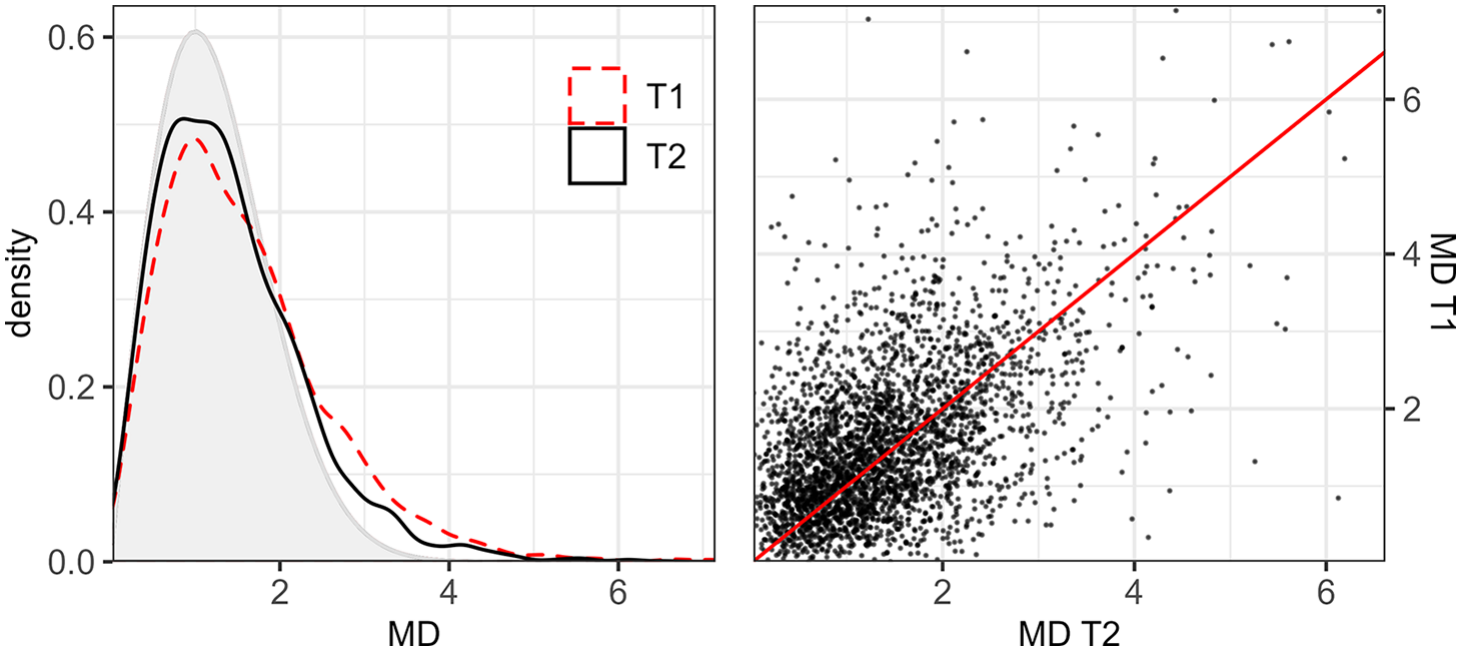
On the left, density plot of empirical Mahalanobis distances (MDs) at T1 (red dashed) and T2 (black solid), compared to a theoretical χ_2_ distribution (gray); on the right, scatter plot of distances at T1 versus T2.

As mentioned above, two general trends emerge in spite of the observed variability: there has been more change than stability over time, and there has been more convergence than divergence, which will now be explored quantitatively. Our quantitative analysis is based on Mahalanobis distances (MDs in the following figures) between the tokens produced by the mobile speakers and the centroids of those produced by the sedentary speakers relative to the latter’s distributions. Since MDs are not widely used in phonetic sciences, we felt important, as a first step, to show through Figure 10 how raw data are distributed and the range of values they cover. The left panel consists of a density plot of the empirical data at T1 (red dashed) and T2 (black solid). The gray-shaded area represents the curve that would be obtained if the tokens of the mobile speakers were distributed in the same way as those from the sedentary speakers (χ_2_ distribution). We observe that the vast majority of the raw distances fall within the 0 to 4 range. The empirical curves are also more right-skewed, and the T2 curve is closer than the T1 curve to the gray one, suggesting a reduction of MD values over time. The right panel consists of a scatter plot of empirical distances at T1 versus T2 (paired by speaker, word, and repetition, e.g., /ʊ/ from the third repetition of word *soupe* by Mobile01 at T1 vs. T2). The data are positively correlated, and as evidenced by the 45° reference line, MD values tend to be more frequently reduced over time than the other way around.

**Figure 11.**
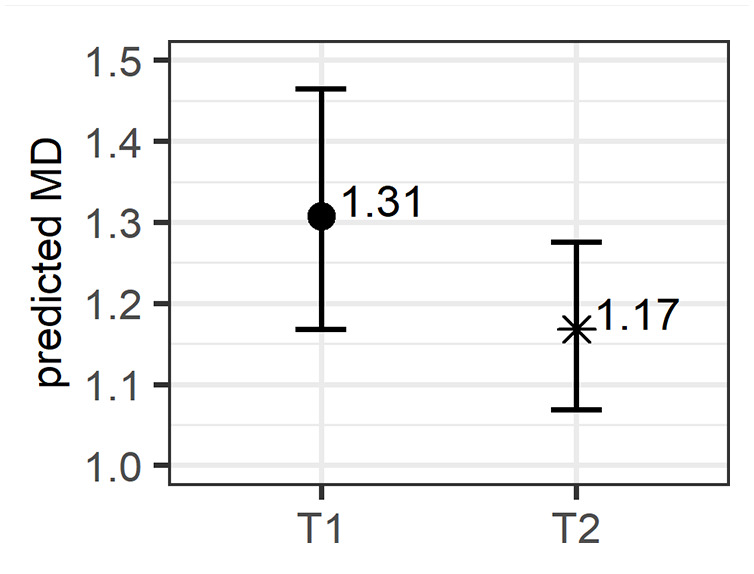
Predicted Mahalanobis distance (MD) over time (log-values from the model have been exponentiated).

The output of the model fit to the log-transformed MDs is shown in Table 2. The two-level fixed factor *Time* has a significant effect on the response variable, that is, distances change over time. As further illustrated in Figure 11, which displays the estimates from the model transformed back into MDs, lower values are predicted at T2 than T1. The average distance is estimated to have gone down from 1.31 at T1 to 1.17 at T2.

**Table 2.** Output of the linear mixed-effects regression model in (1).

*Predictors*	**log-MD**				
	*Estimates*	*SE*	*CI*	*Statistic*	*p*
(Intercept)	0.27	0.06	0.15 to 0.38	4.64	**<.001**
Time (T2)		0.03	−0.17 to −0.05	−3.62	**<.001**
**Random Effects**					
σ^2^	0.46				
τ_00 Speaker_	0.03				
τ_00 Vowel_	0.02				
τ_11 SpeakerTimeT2_	0.00				
τ_11 Vowel.TimeT2_	0.01				
ρ_01 Speaker_	−0.57				
ρ_01 Vowel_	−0.88				
ICC	0.07				
*N*_Speaker_	15				
*N*_Vowel_	14				
Observations	6,262				
Marginal *R*^2^/Conditional *R*^2^	.006/.081				

Estimates are reported on the logarithmic scale. MD = Mahalanobis distance; ICC = intraclass correlation coefficient; CI = confidence interval; *SE* = standard error.

Table 2 additionally shows that the correlation between the random intercept and slope is negative for both Speaker (−0.57) and Vowel (−0.88). Such strong negative correlations indicate that the change from T1 to T2 in the direction of the targets was large when the initial distance was large, but smaller when the initial distance was small.

**Figure 12.**
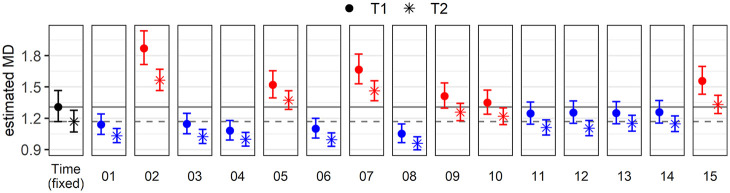
Exponentiated estimated Mahalanobis distances (MDs) at T1 (dot) and T2 (asterisk) for each individual speaker (the 15 levels of the random effect Speaker). As a reference, the estimates for the fixed effect Time are shown on the leftmost panel and reproduced in each panel as solid (T1) and dashed (T2) lines.

We have further explored estimates per Speaker at T1 and T2, which indicate how far or close to the global trend individual participants are (see Drager & Hay, 2012). Figure 12 shows how estimated MDs vary from one speaker to the other, and between T1 and T2. Six out of 15 participants have higher estimates at T1 than that of the fixed effect (red in the electronic version of the paper), meaning that they are further away from the centroids than average. The remaining nine participants were closer than average to the centroids at T1 (blue), with smaller estimates than that of the fixed effect. The estimate of Mobile02 deviates the most from the global trend, with particularly large estimated MDs at T1. Mobile05, whose unusual vowel system has been discussed above (see Figure 9), also has larger estimated MDs than the global trend, but does not stand out as much as Mobile02. This trend could reflect that Mobile02 was more systematic in being far away from the targets at T1 than Mobile05.

Figure 12 also shows estimated MDs at T2 per speaker (asterisks). All differences between T1 and T2 are negative (the asterisk is lower than the dot in each panel), meaning that the estimated distance to the centroid diminishes over time for all speakers, some slightly more than average (e.g., Mobile02) and some slightly less (e.g., Mobile13). Note, however, that although we reproduce these results here, the random slope of Time per Speaker explains very little variance; it turns out to be the only non-significant parameter in the model. We can infer that the random effect of the speaker is quite constant across time.

**Figure 13.**
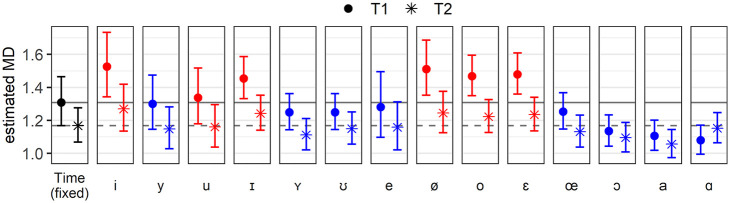
Exponentiated Mahalanobis distances (MDs) at T1 (dot) and T2 (asterisk) of each vowel (the 14 levels of the random effect Vowel). As a reference, the estimates for the fixed effect Time are shown on the leftmost panel and reproduced in each panel as solid (T1) and dashed (T2) lines.

Estimates per Vowel at T1 and T2 are presented in Figure 13. Six have higher estimates at T1 than the fixed effect (red in the electronic version), meaning that the mobile speakers’ productions of these vowels at T1 were farther away from the centroids than average, while the opposite is true for the remaining eight vowels (blue). Regarding estimates at T2, only that of /ɑ/ is higher than at T1: it is the only vowel for which the estimated MD has increased over time, as reflected by the fact that the asterisk is higher than the dot for /ɑ/ in Figure 13. All the other vowels have smaller estimated MDs at T2 than T1. Larger distances at T1 (higher dots) clearly diminish more over time than smaller distances (lower dots), reflecting the strong negative correlation between the random intercept and slope displayed in Table 2 (−0.88). We do not observe any particular trend with respect to the identity of the vowels; for example, there is no such tendency for, say, high vowels to deviate more from the trend, or to only have larger estimated distances.

**Figure 14.**
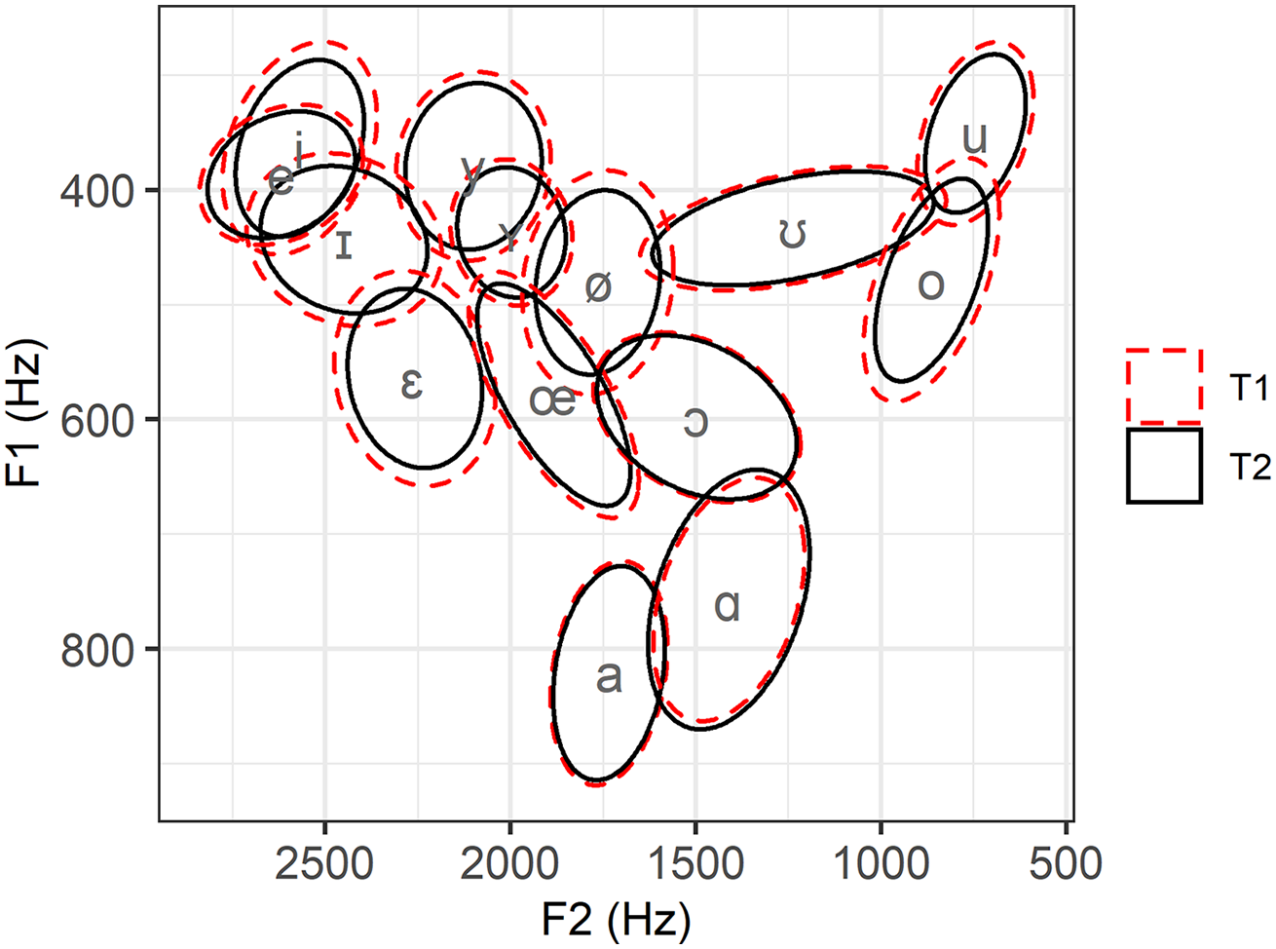
Estimated average Mahalanobis distances away from the centroids (phonetic symbols) at T1 (red dashed ellipses) and T2 (black solid ellipses) in an F1 × F2 plane.

Estimates per Vowel were also used to recreate an F1 × F2 plane where estimated MDs at T1 and T2 are represented by ellipses. In Figure 14, the red dashed ellipses correspond to the estimated mean MD from the centroid at T1 for each vowel. The black solid ellipses correspond to the estimated mean MDs to the centroid at T2. Figure 14 brings new information regarding convergence, clearly reflecting that the value of the metric depends on the distribution based on which it is calculated. For instance, /ɑ/ has fairly large ellipses compared to the other vowels, yet it has the smallest T1 estimate in Figure 13, which is due to the fact that the distribution of the data from the sedentary speakers is quite spread (see Figure 3 and Appendix C). Most importantly, we observe that the largest reduction of MDs over time tends to take place in vowels with many immediate competitors in the F1 × F2 plane, that is, where the vowel space is most crowded. By contrast, in the low vowel area, for instance, /a/ and /ɑ/ are fairly well separated from their closest competitors and have only given way to very reduced change over time. That said, the magnitude of the longitudinal changes for individual vowels appears quite small when the full scale of the vowel space in considered.

This second section on results has focused on MDs, specifically on the results of a linear mixed-effects regression model where they were set as the response variable, Time as a fixed factor, and Speaker and Vowel as random factors with random intercepts and slopes for Time. We have found that MDs were reduced over time, indicating overall convergence by the mobile speakers toward the vowel system of the sedentary speakers. In addition, estimates per Speaker suggested the vowel system of Mobile02 was singularly distant from the target system, but that all in all, the random effect of the speakers was constant across time. Random estimates per Vowel showed that convergence occurred for all vowels but /ɑ/. When the estimated initial distance was large, that is, for /i u ɪ ø o ɛ/, then convergence was large, while change was smaller when the initial distance was small, that is, for /y ʏ ʊ e œ ɔ a ɑ/ (see Figure 13). It is not clear which acoustic criteria could account for these two groups of vowels, but note that we only considered F1 and F2 at the midpoint of vowels classified into broad phonemic categories. Projecting back these estimates onto an F1 × F2 plane suggested convergence was in general of small magnitude, but also tended to be more marked in crowded areas of the vowel space.

## 5 Discussion

This article revolves around SDA and phonetic convergence, two processes that see speakers variably adapt their speech to that of interlocutors. While previous work on SDA has typically focused on large and noticeable phonetic differences, some studies on convergence suggest that the changes taking place in mobile speakers may be more subtle and fine-grained. We addressed this issue by setting up a longitudinal experiment and using a metric that could reveal incremental changes occurring in early stages of SDA. We chose to do so in Quebec French, which allowed to investigate SDA in a relatively crowded vowel space.

Fifteen mobile and eight sedentary female participants were recruited at Université Laval, in Quebec City (Quebec, Canada). Reading tasks featuring 70 phonemic monophthongal oral vowels were completed twice by the mobile speakers (T1 and T2) and once by the sedentary speakers (T2). The first two formants were measured at vowel midpoint; from there, we first built a target vowel system constituted of data from the sedentary speakers. We compared qualitatively, but also quantitatively using Mahalanobis distances, how far from the target system the mobile speakers were at T1, then at T2.

Already at T1, the acoustic characteristics of the mobile speakers’ vowels were visually not drastically different from those of the sedentary speakers (see Appendix D). Despite this, change happened over time, and distance to the target system was significantly reduced. The statistical analysis that was carried out revealed small but recurrent convergence, both across vowels and speakers. This tends to suggest that SDA is not limited to large and noticeable phonetic differences, which has methodological implications for this field of study. Future research on SDA could definitely benefit from being more frequently longitudinal in nature and from having less priors regarding which phonetic features may or may not change, both common practices in convergence studies.

We have also observed that the more crowded the vowel space, the more marked convergence seemed to be. In contrast, where distinctions between vocalic categories were coarser, in particular in the area of the low vowels, there was either very small convergence (/a/) or very small divergence (/ɑ/). Greater convergence amid crowdedness supports the idea of contrast preservation (Lindblom, 1986; Manuel, 1990; Nielsen, 2011; Wagner et al., 2021), even within subsets only of a vocalic system. It also suggests that with more acoustic competitors came more sensitivity on the part of the speakers, whose increased attunement to very small variations where relevant made them fit to finely adjust their productions toward the targets. When vocalic categories were acoustically more distant, the mobile speakers were not as sharply tuned to the targets. If this hypothesis holds, we may infer that the acoustic specificities of a given language or variety are highly determinative of the changes mobile speakers will experience during SDA. As a matter of fact, in an influential paper on the linguistic mechanisms underlying SDA, Chambers (1992) argued that acquisition was more difficult when linguistic rules in the second dialect were more complex than in the first, but that if complexity decreased or stayed the same, then acquisition was more likely (Principles 3 and 7). This could also account for our results and interpretation. Because the mobile speakers’ original vowel space had similar areas of density in the first place, they were in a position to adapt to the equally dense and complex areas of the vowel space of Quebec City, despite the fact that some of their productions already fell within a local range upon arrival.

These small but non-random changes are very much compatible with exemplar-based models, which as mentioned in the Introduction have been put forward in recent work on SDA (e.g., Love & Walker, 2013; Nycz, 2013; Reubold & Harrington, 2018; Rys et al., 2017; Walker, 2018). Indeed, such models posit a continuous upgrading and updating of the speakers-listeners’ categories where fine-grained acoustic properties are instrumental. Their advent marked a break with prior work that interpreted SDA within the accommodation framework (Giles, 1973; Giles et al., 1991; Trudgill, 1986), for which this kind of small changes were not a major concern. Their focus was on communicational strategies put in place by a speaker to balance out a specific interaction, which of course our participants are not immune to, and in that, our results do not provide evidence *against* accommodation theories, but they can more readily be accounted for by exemplar models.

The conceptual shift from these one-off strategies to long-lasting changes was devised by Trudgill (1986), who suggested that accumulated acts of short-term accommodation led to long-term accommodation, or SDA. The author likely envisioned this as repeatedly accommodating on large and noticeable phonetic differences, eventually leading to a more permanent change. This is speculative, but given the small changes we have observed in the very early stages of SDA, we are tempted to extend the idea and infer that perhaps, accumulating convergence on small acoustic distances may result in a large acoustic change. SDA could be incremental in terms of number of acts of convergence, but also *size* of the acts of convergence, at least for non-discrete features like vowel quality. This would further bridge the gap between exemplar- and accommodation-based explanations of SDA, which are in any case more complementary than contradictory (Coles-Harris, 2017).

While we mainly measured convergence, we observed another recurrent qualitative profile of change: vocalic space compression (see Figures 8 and 9). Vocalic space compression typically brings categories closer to one another, increasing overlap and reducing contrasts. It has been attributed to reduced articulatory movements, themselves caused by changes in constraints of the communication situation (Lindblom, 1990; Smiljanić & Bradlow, 2009). Given that no modification was brought to the speech production tasks and global experimental protocol at T2 (Section 3.2), one possible explanation for this profile of change might be that the speakers were not confronted with novelty anymore. Going over a known protocol with a researcher they had interacted with before might have changed the way they apprehended the communication situation and led to vocalic space compression. If this was the case, overcoming this would constitute an interesting challenge for researchers looking to collect longitudinal speech data (see Wagner & Tagliamonte, 2018).

MDs have proven to be a powerful tool that could be more widely used in studies on SDA. They capture a centroid, but also a distribution, making them more sensitive to important aspects of the acoustic space than Euclidean distances. Above all, they allow to embrace the typical yet still puzzling heterogeneity of SDA. Our data is a very good test of MDs’ capacity to cope with variation, not just because it is about SDA, but also because the unit we assessed change from, that is, vowels, contains several sources of variability. For instance, when they appear in certain consonantal contexts, some vowels can be diphthongized to a certain degree by some Quebec French speakers (see Paradis & Dolbec, 1998 and references therein); when this is the case, these contextually diphthongized vowels rise in the acoustic space (Dumas, 1974). This may result in increased variability in F1 measurements taken at vowel midpoint, but the computed MDs will compensate for this inasmuch as both mobile and sedentary datasets are affected by this source of variability: more scattered mobile tokens will be measured against a more elongated sedentary distribution. This compensation would not happen with classic Euclidean distances to the centroids. Similarly, there are coarticulatory effects from the preceding and following consonants that are not necessarily balanced across vowel categories (but constant across speakers). Furthermore, our strategy for dealing with possible biases induced by a specific reading task has been to dilute them with biases from two other tasks, which, ultimately, serves its purpose, but also induces further variability within vowel categories. Despite all this, MDs allowed coherent and interpretable trends to emerge, highlighting their possible utility in other research fields where variability is pervasive, for example, SLA (Kartushina et al., 2015; Melnik-Leroy et al., 2021; Tomaru & Arai, 2019), speech errors (Marin et al., 2010; Pouplier et al., 2014), sound change (Harrington & Schiel, 2017; Siddins & Harrington, 2015), and work involving spontaneous speech more generally. Further advantages are that they can be obtained from other types of data for which Gaussianity can be assumed, and are not limited to bi-dimensional spaces. We do not know, however, whether MDs are in any way representative of human perception. This could be verified through a perception experiment that could simultaneously allow us to verify whether the measured convergence is actually perceptible to untrained or trained listeners (e.g., Pardo, 2006; Pardo et al., 2012).

In this first study on SDA in Quebec French, we have chosen to focus exclusively on F1 and F2 at vowel midpoint. We are aware that this is a rather simplistic, although robust, parametrization of vocalic identity. Other parameters could have been utilized, in particular formant dynamics, given that phonetic diphthongization is common in some contexts in Quebec French as mentioned above (Dumas, 1974, 1987; Leblanc, 2012; Riverin-Coutlée & Roy, 2020; Santerre & Millo, 1978; Walker, 1984; etc.), and also that formant dynamics may contribute to maintaining contrasts in crowded vowel spaces (Peters et al., 2017; see also Nearey & Assmann, 1986). This is a limitation to our study that could launch future work with tremendous potential.

Other limitations include sample size, a typical issue of longitudinal work (Sankoff, 2018a). We had originally recorded 24 mobile speakers at T1, but only 15 returned at T2 and are part of this study (37.5% sample attrition in just 1 year). Moreover, we are aware that our corpus cannot be considered representative of Quebec French, as 15 female university students are not representative of all Quebec French speakers or all mobile speakers, and the reading tasks do not reflect all the communication situations a speaker may encounter in their daily life. Furthermore, all vowels produced in all consonantal contexts have been treated equally, but we cannot exclude that some of them may be more prone to convergence, potentially those that vary regionally (Babel, 2012), which could be explored in future work targeting mobile speakers with a more homogeneous geographic background.

In many SDA studies, extra-linguistic factors are given much weight. As briefly mentioned in the Introduction, factors like social integration, age, identity, and attitudes have sometimes been found to influence SDA. To give just one example, Pesqueira (2008) showed that some features of Mexican Spanish tended to be more readily acquired by mobile Argentineans whose spouse and social network were Mexican and who planned on staying in Mexico, compared to mobile speakers who kept strong social ties with Argentineans and planned on returning to Argentina. Bringing qualitative information of this kind into play may help explain heterogeneity and behaviors such as lack of use of the second dialect or divergence, but not having done so in the current study does not mean that the issue was simply overlooked. The truth is, work remains to be done before questionnaires and scales that are sensitive and gradated enough to account for factors as complex as social integration in a new city can be effectively used (but see Walker, 2014, 2018 for an attempt). Since, to this day, we do not have at our disposal robust quantitative indexes that can be included into statistical models, we chose to put aside extra-linguistic factors and focus on linguistic factors instead, at least for now.

Another aspect of this study we wish to discuss is that we have exclusively looked at university students. Alongside the usual ease-of-recruitment argument, this population presents sensible advantages when addressing SDA. As Nycz (2015) points out, it can be extremely difficult to locate newcomers in the general population and to record mobile speakers shortly before or after they move, whereas with every semester come new students. Finding mobile speakers with similar life trajectories to form a coherent sample is also a daunting task when looking in the general population. University students are typically more or less the same age and their lives have so far revolved around high school attendance, friendships with peers, living with their parent(s), and limited work experiences. In addition, they stay at least 3 years in the city where they go to university, allowing for longitudinal experiments. These are probably some of the reasons why in the growing body of literature on SDA, an increasing number of studies involve university students (i.e., Bigham, 2010; Campbell-Kibler et al., 2014; De Decker, 2006; Evans & Iverson, 2007; Pardo et al., 2012; Wagner, 2012). Despite numerous methodological differences with our own work and the fact that all these studies investigate dialects of English, one common finding is that a very short time span is necessary for changes to be observed in the speech of university students. For example, Pardo et al. (2012) found some changes over the first 5 months that roommates had lived together, while Evans and Iverson (2007) reported larger differences 2 years after their participants started university, although some changes were already incipient after 3 months. Bigham (2012) has advanced that this population could be especially inclined to rapid linguistic changes that parallel those in other spheres of their lives (Arnett, 2000), although this idea remains to be confirmed by thorough empirical work.

Finally, for practical reasons, the speech of eight sedentary speakers was treated as a target toward which the mobile speakers were to converge as a result of their stay in Quebec City. However, beyond the advantages of studying SDA in universities, Nycz (2015) highlights that it can also be tricky in many ways, not the least being the students’ actual input. As a matter of fact, campuses are linguistically diverse and by no means restricted to local speech. Newcomers looking to build a new social network are likely to meet other newcomers in the same situation, and therefore, be extensively exposed to the speech of speakers from other regions and not so inclined to adopt local practices. This raises further questions about the sedentary students. They, after all, are also exposed to a diverse linguistic environment they may not be impermeable to. Even if we assume they are the ones the mobile speakers will be adapting to, what if they are a moving target?

## 6 Conclusion

Our study on SDA in Quebec French has illustrated that mobile speakers have converged toward the vocalic system of sedentary speakers within a timeframe of just a year, although their productions were not immensely different in the first place and there were several sources of variability in the data. Changes were small but not random, and even appeared closely aligned on specificities of the vowel space. These results show the interest of a greater degree of detail when studying SDA, in line with core ideas put forward within exemplar-based models.

It should be clear, however, that we have only captured an early stage of SDA. As they spend more time in Quebec City, the mobile speakers will likely pick up on other aspects of the local speech and proceed to further adjustments. In this regard, Mobile03 is a good example as her second year in Quebec City did not bring stability. Actually, we were only offered a small window on our participants’ rich linguistic trajectories. In addition to increasingly adapting to Quebec City features, the speakers may also experience further mobility and phonetic changes later in their lives, and some sort of reversion, for example, if returning to their hometown upon completion of their degree, which cannot be excluded either (see Reubold & Harrington, 2018; Shapp et al., 2014).

SDA is a relatively recent field of study that has arisen as geographic mobility increased, but its fast expansion also constitutes a testimony of a paradigm shift in the way the adult speakers’ linguistic system is seen. A growing body of literature emphasizes the dynamic nature of language use throughout the lifespan (Hazan, 2017). In SDA studies like ours, mobile speakers respond to changes in their linguistic environment. In a parallel manner, sedentary speakers have proven sensitive to changes at the community level (e.g., Bowie, 2015; Harrington et al., 2000, et seq.; Sankoff & Blondeau, 2007) and to interactions in a social vacuum (Harrington et al., 2019; Sonderegger et al., 2017). Our results thus add to this shift toward uncovering the extent of adult speakers’ phonetic flexibility.

Finally, our study was only possible by comparing the mobile speakers with themselves, that is, by measuring their evolution from a baseline. Such longitudinal studies are not the easiest. The longer the time span covered, the more obstacles arise. Participants drop off or cannot be reached, research teams are renewed (at best), tools and methods change, and individual life trajectories become less and less comparable (see Sankoff, 2018a, 2018b). Despite these downsides, panel studies should not be as exceptional as they are today (Hazan, 2017), given that they offer unprecedented insights into the mechanisms of language variation and change, at both the community and the individual levels.

## Supplemental Material

sj-csv-1-las-10.1177_00238309221097978 – Supplemental material for Using Mahalanobis Distances to Investigate Second Dialect Acquisition: A Study on Quebec French Supplemental material, sj-csv-1-las-10.1177_00238309221097978 for Using Mahalanobis Distances to Investigate Second Dialect Acquisition: A Study on Quebec French by Josiane Riverin-Coutlée, Johanna-Pascale Roy and Michele Gubian in Language and Speech

sj-pdf-1-las-10.1177_00238309221097978 – Supplemental material for Using Mahalanobis Distances to Investigate Second Dialect Acquisition: A Study on Quebec French Supplemental material, sj-pdf-1-las-10.1177_00238309221097978 for Using Mahalanobis Distances to Investigate Second Dialect Acquisition: A Study on Quebec French by Josiane Riverin-Coutlée, Johanna-Pascale Roy and Michele Gubian in Language and Speech

## Figures and Tables

**Figure C1.**
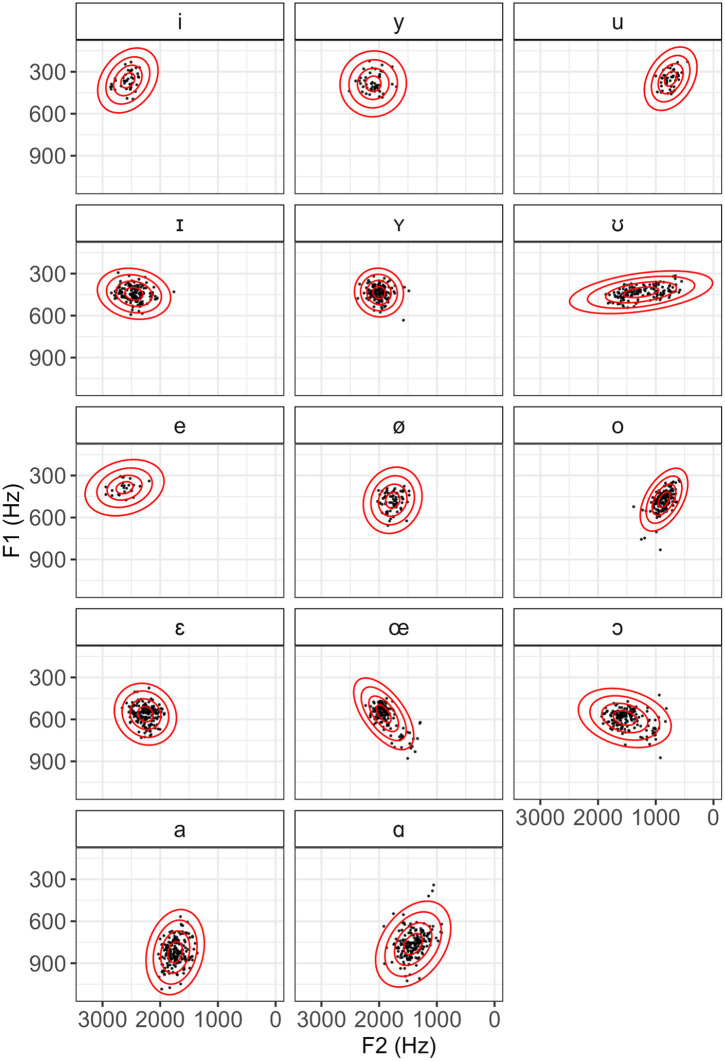
Tokens produced by the sedentary speakers in F1 × F2 planes. The four ellipses in each panel correspond to 1, 2, 3, and 4 Mahalanobis distances away from the centroid of each distribution.

**Figure D1.**
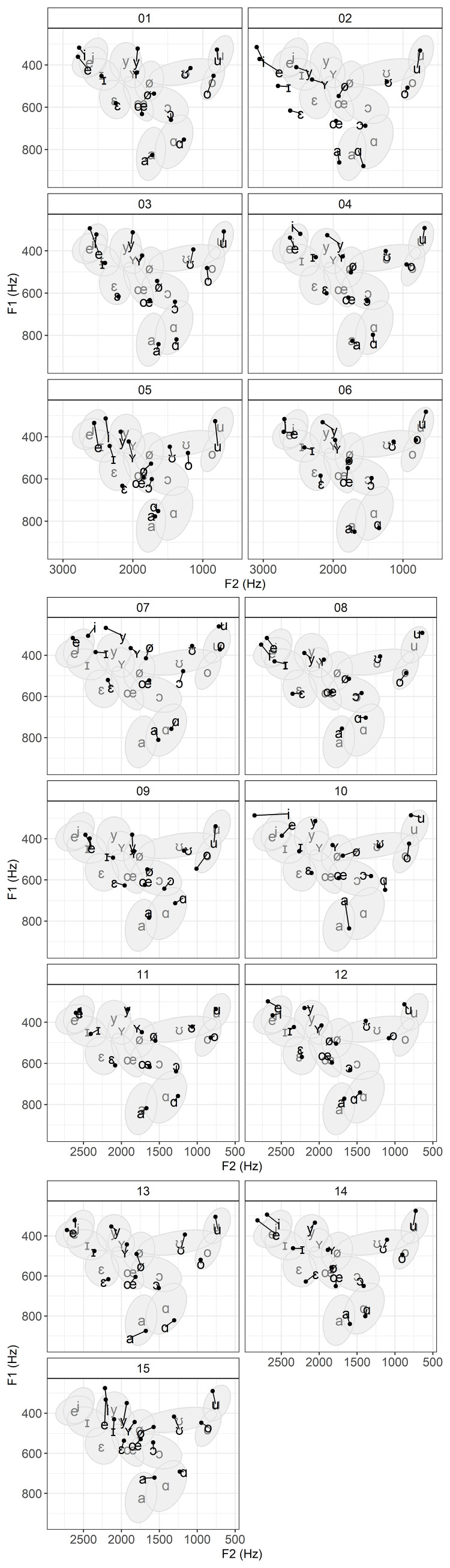
Mobile speakers’ individual trajectories over time in F1 × F2 planes. The gray vowels and ellipses correspond to the target system (sedentary speakers). The black phonetic symbols connected to the dots are the mobile speakers’ mean productions at T2 and T1, respectively.

## Appendix A

**Table A1.** Words featuring stressed monophthongal oral vowels produced by the participants.

/i/	vie (n) “life”	chétive (a) “sickly”		
/ɪ/	frite (n) “(French) fry”friche (n) “fallow”	figue (n) “fig”frime (n) “sham”	désir (n) “desire”	pile (n) “battery”
/y/	déchu (a) “deposed”	fuse (v) “fly”		
/ʏ/	flûte (n) “flute”puce (n) “flea”	élude (v) “avoid”	pur (a) “pure”	cellule (n) “cell”
/u	fou (n) “crazy”	rouge (a) “red”		
/ʊ/	soupe (n) “soup”débouche (v) “lead to”	soude (v) “weld” gougoune (n) “thong”	four (n) “oven”	houle (n) “swell”
/e/	aller (v) “to go”			
/ø/	vœu (n) “wish”	creuse (a) “deep”	jeûne (n) “fast”	
/o/	peau (n) “skin”drôle (a) “funny”	rauque (a) “hoarse”fosse (n) “pit”	aube (n) “dawn”jaune (a) “yellow”	chose (n) “thing”
/ɛ/	passait (v) “passed by”belle (a) “beautiful”	bec (n) “beak”crèche (n) “manger”	plaide (v) “plead”sème (v) “sow”	brève (a) “brief”
/œ/	le (p) “it”œuf (n) “egg”	beurre (n) “butter”jeune (a) “young”	neuve (a) “new”	veulent (v) “(they) want”
/ɔ/	bloc (n) “block”école (n) “school”	blogue (n) “blog”bosse (n) “bump”	porc (n) “pig”pomme (n) “apple”	toge (n) “toga”
/a/	fa (n) “F (music note)”page (n) “page”	flaque (n) “puddle”pédale (v) “pedal”	blague (n) “joke”face (n) “face”	prépare (v) “prepare”femme (n) “woman”
/ɑ/	état (n) “state”châle (n) “shawl”	Pâques (n) “Easter”lâche (v) “release”	part (n) “ (on their) part”âne (n) “donkey”	

Translations reflect meanings induced by carrier sentences (e.g., *Fais-* ***le*** “Do **it**”). Some words or their meanings are specific to Quebec French (e.g., *gougoune* “thong”). Grammatical categories following the words go as follows: (n) noun, (v) verb, (a) adjective or past participle acting as an adjective, (p) pronoun.

## Appendix B

We computed the Mahalanobis distance (MD) of all tokens produced by the mobile speakers relative to the distribution of the tokens produced by the sedentary speakers in the F1 × F2 space, for each vocalic category separately, with the equation presented in (2)

(2)
$$MD\left(x\right)=\sqrt{{\left(x-\mu \right)}^{T}C^{-1}(x-\mu )}$$

where x is a vector of two formant values for a token, µ is a vector of the coordinates of the centroid of the distribution, and C is the 2 × 2 covariance matrix.

The encompassed probability masses at integer MDs vary depending on the number of dimensions considered (two in our case). In general, the MDs of datapoints belonging to a multidimensional Gaussian distribution from its centroid follow a $\chi_{d}$ (chi) distribution of degrees of freedom *d* equal to the number of dimensions. Given a MD and a *d*-dimensional Gaussian, the encompassed probability mass within an ellipsoid centered at the mean is $F_{\chi_{d}}(MD)$ , where F() indicates the cumulative distribution function F(y)=Prob(x≤y) .

In the two-dimensional case, these probability masses amount to the percentages reported in Section 3.3 in relation to Figure 2. In the one-dimensional case, 1 MD encompasses 68.27% of the probability mass, 2 MD 95.45%, 3 MD 99.73%, and 4 MD 99.99%; these percentages coincide with those of *z* scores.

## Notes

1. The concept of salience is notoriously hard to pin down and a consensual, working definition has yet to be laid down (e.g., Auer et al., 1998; Boswijk et al., 2020; Campbell-Kibler, 2016; Watson & Clark, 2013). That said, a salient feature can roughly be described as a phonetically large and sociolinguistically meaningful difference that speakers are aware of. Of course, the size of a phonetic difference is relative.

2. Throughout this article, “Quebec French” will refer to the variety of French spoken in the Canadian province of Quebec. As explained in Section 3.1, some of our participants are from the capital of the province, Quebec City. The speech of Quebec City is neither more nor less representative of Quebec French than that spoken in other cities.

3. The effect of /ʁ/ is subject to debate, some authors considering that it is preceded by the tense variants and others by the lax. Following recent analyses (Côté, 2012; Sigouin & Arnaud, 2015), we consider in this study that /ʁ/ is preceded by the lax variants (e.g., *pur* [pʏʁ] “pure,” *four* [fʊʁ] “oven”).

4. Université Laval has the second-largest student population in Quebec after Université de Montréal (Ministère de l’Éducation et de l’Enseignement supérieur du Québec, 2020). Approximately 87% of the students come from the province of Quebec; among them, about half of the undergraduate students enrolling each year come from the Quebec City area, and the other half, from other regions across Quebec (Commission des affaires étudiantes de l’Université Laval, 2016). The mobile speakers in this study came from Amos, Contrecœur, Dolbeau-Mistassini, Plaisance, Rimouski (2), Saguenay, Sherbrooke, Sainte-Anne-des-Monts, Saint-Élie-de-Caxton, Saint-Léonard-d’Aston, Trois-Rivières, Val-d’Or and Victoriaville (2).

5. See also Cohen Priva and Sanker (2018, 2019) and MacLeod (2021) regarding limitations of the Euclidean distances in convergence studies and a suggestion for an alternative analysis technique.

6. This was done via the coef() function from the R *stats* package (see supplementary material).
