# Supplementary material for: Using Mahalanobis Distances to Investigate Second Dialect Acquisition: A Study on Quebec French
Source: Lang Speech. 2022 Jun 20;66(2):291–321. doi: 10.1177/00238309221097978 (PMC10230596; doi:10.1177/00238309221097978)
Supplement: sj-pdf-1-las-10.1177_00238309221097978 – Supplemental material for Using Mahalanobis Distances to Investigate Second Dialect Acquisition: A Study on Quebec French [file sj-pdf-1-las-10.1177_00238309221097978.pdf]

```r
# Libraries ----
library(tidyverse)
library(emmeans)
library(ggforce)
library(lme4)
library(lmerTest)
library(magrittr)
library(sjPlot)
library(dplyr)


# Set working directory ----
setwd("")

# Read data ----
data = read.csv(file = "LAS200055_data.csv")

# Compute Mahalanobis distances ----

# 1) reference Gaussians from sedentary
mahalRef = data %>%
  filter(Status %in% "Sedentary") %>%
  group_by(Vowel) %>%
  summarise(Mean = cur_data() %>%
              select(F1_50, F2_50) %>%
              colMeans() %>%
              list(),
            Cov = cur_data() %>%
              select(F1_50, F2_50) %>%
              cov() %>%
              list())

# 2) compute Mahalanobis distance of mobile speakers' tokens with respect to reference (sedentary) distributions
mahalMobile = data %>%
  filter(Status %in% "Mobile") %>%
  inner_join(mahalRef, by = "Vowel") %>%
  group_by(Vowel, Speaker, Token) %>%
  mutate(maha.score = mahalanobis(c(F1_50, F2_50), Mean[[1]], Cov[[1]]) %>% sqrt())



# Statistical analysis ----

# log-transform Mahalanobis distances
mahalMobile = mahalMobile %>%
  mutate(log.maha = log(maha.score))

# run lmer model
m1 = lmer(log.maha ~ Time + (Time|Speaker) + (Time|Vowel),
          control=lmerControl(optCtrl=list(xtol_abs=1e-7, ftol_abs=1e-7)),
          data = mahalMobile)

# summary of results
summary(m1)
# summary of results in a table (corresponds to Table 2)
tab_model(m1,
          show.se = TRUE,
          show.stat = TRUE,
          dv.labels = "log-MD")



# Extract and plot fixed effect ----

# Only intercept and slope of fixed effect (logarithmic scale)
fixef(m1)

# Estimates at T1 and T2 on original MD scale (exponentiation)
```

```
m1.plot.fixed = get_model_data(m1, type = c("pred"), terms="Time") %>%
  rename("Time" = "x") %>%
  mutate(Time = recode(Time, "1" = "T1", "2" = "T2"),
         pred_exp = exp(predicted),
         exp_low = exp(conf.low),
         exp_high = exp(conf.high),
         pred_lab = round(pred_exp, 2))

# Plot estimates for fixed effect Time (corresponds to Figure 11)
m1.plot.fixed %>%
  ggplot() +
  aes(y=pred_exp, x=Time) +
  geom_point(aes(shape=Time),
             size=2,
             show.legend = FALSE) +
  scale_shape_manual(values=c(19,8)) +
  geom_text(aes(label=pred_lab),
            hjust=-.2,
            vjust=0,
            size=3) +
  geom_errorbar(aes(ymin=exp_low, ymax=exp_high, x=Time),
                width=.2) +
  scale_x_discrete(name=NULL) +
  scale_y_continuous(name="predicted MD",
                     limits=c(1, 1.5)) +
  theme_bw() +
  theme(axis.text=element_text(size=8),
        axis.title=element_text(size=8))


# Extract and plot random effects ----

# Estimated marginal means corrected for random effects for each speaker and vowel across time (logarithmic scale)
coef(m1)

# extract standard deviation for estimates of Vowel and Speaker (logarithmic scale)
sdrandef = as.data.frame(ranef(m1))

# function to extract estimates & sd for random effects and exponentiate
prepare_rand_for_plot <- function(coef_df, sdrandef, m1.plot.fixed) {
  coef_df %>%
    rownames_to_column(var = "grp") %>%
    mutate(TimeT2 = `(Intercept)` + TimeT2) %>%
    mutate(abovebelow = ifelse(`(Intercept)` > pull(m1.plot.fixed %>%
                                                     filter(Time %in% "T1") %>%
                                                     select(predicted)), "above", "below")) %>%
    pivot_longer(cols = c(`(Intercept)`, TimeT2),
                 names_to = "term",
                 values_to = "coefficients") %>%
    inner_join(sdrandef, by = c("term", "grp")) %>%
    mutate(coefficients_exp = exp(coefficients),
           min.sd_exp = exp(coefficients - 2 * condsd),
           max.sd_exp = exp(coefficients + 2 * condsd)) %>%
    mutate(term = case_when(term == "(Intercept)" ~ "T1",
                            term == "TimeT2" ~ "T2")) %>%
    bind_rows(m1.plot.fixed %>%
                tibble() %>%
                select(Time, pred_exp, exp_low, exp_high) %>%
                rename(term = Time,
                       coefficients_exp = pred_exp,
                       min.sd_exp = exp_low,
                       max.sd_exp = exp_high) %>%
                mutate(grp = "Time\n(fixed)",
                       abovebelow = "equal"))
}
```

```r
# apply function to random effect Vowel
randef.V = prepare_rand_for_plot(as.data.frame(coef(m1)$Vowel), sdrandef, m1.plot.fixed) %>%
  mutate(grp = factor(grp, levels=c("Time\n(fixed)", "i", "y", "u", "I", "Y", "U", "e", "2", "o", "E", "9", "O", "a", "A")))


# apply function to random effect Speaker
randef.S = prepare_rand_for_plot(as.data.frame(coef(m1)$Speaker), sdrandef, m1.plot.fixed) %>%
  mutate(grp = recode(grp, "1" = "01", "2" = "02", "3" = "03", "4" = "04", "5" = "05", "6" = "06", "7" = "07", "8" = "08", "9" = "09")) %>%
  mutate(grp = factor(grp, levels=c("Time\n(fixed)", "01", "02", "03", "04", "05", "06", "07", "08", "09", "10", "11", "12", "13", "14", "15")))

# plot random effect Speaker (corresponds to Figure 12)
# the same can be done with randef.V for random effect Vowel (corresponds to Figure 13, but with SAMPA symbols)

randef.S %>%
  ggplot() +
  aes(x=term, y=coefficients_exp, group=term, col=abovebelow) +
  facet_wrap(~grp, nrow=1, strip.position = "bottom") +
  geom_hline(yintercept = m1.plot.fixed %>% tibble() %>% filter(Time == "T1") %>% pull(pred_exp),
             colour="gray50", linetype=1) +
  geom_hline(yintercept = m1.plot.fixed %>% tibble() %>% filter(Time == "T2") %>% pull(pred_exp),
             colour="gray50", linetype=2) +
  geom_point(size=2, aes(shape=term)) +
  geom_errorbar(aes(ymin=min.sd_exp,
                    ymax=max.sd_exp), width=.5) +
  scale_x_discrete(name=NULL, labels=NULL, breaks=NULL) +
  scale_y_continuous(name="estimated MD") +
  scale_shape_manual(values=c(19,8)) +
  scale_colour_manual(values=c("red", "blue", "black"),
                      guide="none") +
  theme_bw() +
  theme(axis.text = element_text(size=10),
        axis.title = element_text(size=10),
        legend.title = element_blank(),
        legend.text = element_text(size=10),
        legend.position = "top",
        legend.margin = margin(b=-.3, unit="cm"),
        strip.background = element_blank())


# Re-create ellipses from random coefficients ----

# 2D ellipses can be projected onto an F1/F2 plane
# because Mahalanobis distances have been calculated based on F1 and F2

# function to get ellipses from covariance matrix
# see e.g. Section 2.3 in Bishop, C. M. (2006). Pattern Recognition and Machine Learning. Springer.
ellipse_from_cov <- function(S, mahal) {
  # `S`: covariance matrix
  # `mahal`: Mahalanobis distance
  # Value: a list:
  # `a`, `b`: ellipse axes
  # `angle`: angle with respect to x axis
  Eig <- eigen(S)
  axes <- sqrt(mahal^2 * Eig$values)
  angle <- atan(Eig$vectors[2,1]/Eig$vectors[1,1])
  list(a = axes[1], b = axes[2], angle = angle)
}


# for the purpose of this demonstration on how to draw an ellipse, we will only select
```

```
[i]
v = "i"

# calculate centroid of vowel categories of sedentary speakers
centroid = data %>%
  filter(Status %in% "Sedentary") %>%
  group_by(Vowel, Speaker) %>%
  summarise(F1_50 = mean(F1_50), F2_50 = mean(F2_50)) %>%
  group_by(Vowel) %>%
  summarise(F1_50 = mean(F1_50), F2_50 = mean(F2_50)) %>%
  ungroup()

# mean vector
m_est = data %>%
  filter(Status %in% "Sedentary" & Vowel %in% v) %>%
  select(c(F2_50, F1_50)) %>%
  as.matrix %>%
  apply(2, mean)

# covariance matrix
s_est = cov(data %>%
              filter(Status %in% "Sedentary" & Vowel %in% v) %>%
              select(c(F2_50, F1_50)) %>%
              as.matrix())


# major and minor axes and angle at T1
ell.i.T1 = ellipse_from_cov(s_est, mahal = pull(randef.V %>%
                                                   filter(Vowel %in% v & term %in%
                                                   "T1") %>%
                                                   select(coefficients_exp))) %>%
  as.data.frame() %>%
  mutate(Time = "T1")

# major and minor axes and angle at T2
ell.i.T2 = ellipse_from_cov(s_est, mahal = pull(randef.V %>%
                                                   filter(Vowel %in% v & term %in%
                                                   "T2") %>%
                                                   select(coefficients_exp))) %>%
  as.data.frame() %>%
  mutate(Time = "T2")

# bind in together
ell.i = rbind(ell.i.T1,
              ell.i.T2)


# plot in F1/F2 plane
ell.i %>%
  ggplot() +
  aes(col=Time, linetype=Time, a=a, b=b, angle=angle) +
  scale_x_reverse(name="F2 (Hz)") +
  scale_y_reverse(name="F1 (Hz)") +
  theme_bw() +
  geom_ellipse(aes(x0 = m_est[1], y0 = m_est[2]), size=.5) +
  scale_color_manual(values=c("red", "black")) +
  scale_linetype_manual(values=c(2, 1)) +
  geom_text(inherit.aes = FALSE, data = (centroid %>% filter(Vowel %in% v)),
            aes(x=F2_50, y=F1_50, label=V),
            size=4, colour="gray40") +
  theme(axis.text = element_text(size=10),
        axis.title = element_text(size=10),
        legend.title = element_blank(),
        legend.text = element_text(size=8),
        legend.margin = margin(0))


# Plot reference distributions ----
# with ellipses at 1, 2, 3, 4 MD from the centroids (corresponds to Figure C1 in
```

```
Appendix C, but with SAMPA symbols)

data %>%
  filter(Status %in% "Sedentary") %>%
  ggplot() +
  aes(x=F2_50, y=F1_50) +
  facet_wrap(~Vowel, nrow=5) +
  scale_x_reverse(name="F2 (Hz)") +
  scale_y_reverse(name="F1 (Hz)") +
  geom_point(size=.1, alpha=.7) +
  stat_ellipse(level=0.3935, colour="red", size=.4) +
  stat_ellipse(level=0.8647, colour="red", size=.4) +
  stat_ellipse(level=0.9889, colour="red", size=.4) +
  stat_ellipse(level=0.9996, colour="red", size=.4) +
  theme_bw() +
  theme(axis.text = element_text(size=12),
        axis.title = element_text(size=12),
        strip.text = element_text(size=12),
        strip.background = element_rect(colour = "black", fill = "white"))
```
